# Efficacy and Safety of Sofosbuvir and Velpatasvir Combination for the Treatment of Chronic Hepatitis C in Patients With or Without Cirrhosis

**DOI:** 10.7759/cureus.19768

**Published:** 2021-11-20

**Authors:** Islam Shah, Wiqas Ahmad, Abdul Qadir, Iltaf Muhammad, Muhammad Islam, Mustaqeem Shah, Naeem Jan, Sadia Anjum

**Affiliations:** 1 Internal Medicine, Khyber Teaching Hospital, Peshawar, PAK; 2 Internal Medicine, Khyber Medical College, Peshawar, PAK; 3 Gastroenterology and Hepatology, Hayatabad Medical Complex, Peshawar, PAK; 4 Internal Medicine, Khyber Medical University, Peshawar, PAK; 5 Gastroenterology and Hepatology, Lady Reading Hospital, Peshawar, PAK; 6 Radiology, Lady Reading Hospital, Peshawar, PAK

**Keywords:** chronic hepatitis c virus, hcv, efficacy, velpatasvir (vel), sofosbuvir (sof)

## Abstract

Background and aim

For years, interferon-based treatment has been offered to patients with chronic hepatitis C virus (HCV) infection; however, the complexity of the treatment, efficacy, and adverse effects were the primary concerns. All these concerns were addressed with the introduction of directly acting antivirals (DAAs) to treat chronic HCV. Sofosbuvir and velpatasvir are second-generation DAAs used in combination for the treatment of chronic HCV infection. The aim of our study was to determine and compare the efficacy and safety profile of the sofosbuvir and velpatasvir combination in treating patients with chronic hepatitis C with or without cirrhosis.

Materials and methods

This descriptive study was conducted at the Department of Medicine, Khyber Teaching Hospital, Peshawar, from March 15th to September 15th, 2021 after approval from the Institution Research and Ethical Review Board (IREB). Diagnosis of HCV was based on the detection of hepatitis C ribonucleic acid (RNA) fragments by reverse transcription-polymerase chain reaction (RT-PCR). Liver status was assessed with liver function tests and imaging. Sofosbuvir (400 mg) and velpatasvir (100 mg) were administered once daily for 12 weeks, followed by polymerase chain reaction (PCR) for HCV RNA after 12 weeks of completion of treatment for determination of sustained virologic response at 12 weeks (SVR12). Patients with cirrhosis also received weight-based ribavirin. Adverse events experienced by the study participants during the course of treatment were recorded. Data were collected regarding patients' demographics, laboratory parameters, SVR12, and adverse events, and were then analyzed using SPSS, version 22 (IBM SPSS Statistics, Armonk, NY).

Results

A total of 58 patients with cirrhosis and 58 patients without cirrhosis with chronic HCV were enrolled. The rate of SVR12 in patients with cirrhosis was 89.7% (52 patients achieved SVR12), compared to 98.3% in patients without cirrhosis (57 patients achieved SVR12). Subgroup analysis of patients with cirrhosis revealed that patients who have failed to achieve SVR12 were mostly males, had prolonged disease duration, and low hemoglobin at baseline; however, the association of these factors with SVR12 was not significant. The incidence of adverse events among all study participants was 46.5%. Among the cirrhotic cohort, 37 (63.8%) patients experienced adverse events, while only 17 (29.3%) patients among the non-cirrhotic cohort had adverse events. A total of 24 patients with cirrhosis (41.37%) reported mild complaints. The most commonly reported adverse event was gastrointestinal (GI) upsets (46.2%), followed by fatigue (33.9%), while 19.9% developed miscellaneous adverse events such as headaches, rash, and insomnia.

Conclusion

The combination of sofosbuvir and velpatasvir is highly effective and safe in patients with HCV with or without cirrhosis. However, this combination's efficacy was slightly higher in non-cirrhotic patients (98.3%) than in cirrhotic patients (89.7%).

## Introduction

Worldwide, Pakistan stands second with respect to the prevalence of hepatitis C. Its prevalence in Pakistan is 4.8%. Lack of population-based large-scale screening drive for occult hepatitis C is rendered as the main reason behind this [1]. Province-wise, the prevalence of hepatitis C is 5.46% in Punjab, 2.55% in Sindh, 6.07% in Khyber Pakhtunkhwa, 25.77% in Baluchistan, and 3.37% in federally administrated tribal areas [2]. Genotype 3a is reported to be the most prevalent [3].

Conventional interferon-based treatment regimen, with or without ribavirin, has been attempted for years to treat chronic hepatitis C; however, the modality did not succeed due to low efficacy, poor treatment schedule, poor compliance, and the associated adverse effects. The introduction of directly acting antivirals (DAAs) for the treatment of chronic hepatitis C was a remarkable milestone. This treatment modality addressed all the concerns related to the conventional treatment regimen, and it is the currently recommended form of treatment in practice [4,5].

Sofosbuvir is a pan-genotypic antiviral that acts by inhibiting hepatitis C virus (HCV) RNA synthesis through inhibition of nonstructural protein 5B (NS5B) polymerase. It possesses fewer adverse outcomes and has a low tendency towards resistance [6]. Velpatasvir is a nonstructural protein 5A (NS5A) HCV protein inhibitor. It also acts against all HCV genotypes [7]. The combination of NS5B inhibitor (sofosbuvir) and NS5A inhibitor (velpatasvir) for the treatment of chronic hepatitis C has shown the efficacy of 99% to 100% (sustained virologic response at 12 weeks [SVR12]) in non-cirrhotic treatment-naïve patients [8]. Not enough work has been done on the efficacy of the sofosbuvir and velpatasvir combination for treating chronic hepatitis C in patients with decompensated liver cirrhosis, nor the safety profile has been evaluated. Therefore, we decided to conduct this study to evaluate the safety and efficacy of the sofosbuvir and velpatasvir combination in our population.

## Materials and methods

Objectives

The purpose of this study was to assess the efficacy and safety profile of the sofosbuvir and velpatasvir combination for the treatment of chronic hepatitis C. Another objective of the study was to compare efficacy and safety profiles among cirrhotic and non-cirrhotic cohorts of patients.

Setting

This descriptive study was conducted at the Department of Medicine, Khyber Teaching Hospital, Peshawar, from 15th March to 15th September 2021, after approval from the Institution Research and Ethical Review Board (IREB) of the Khyber Medical College/Khyber Teaching Hospital (approval number: 670/DME/KMC). Patients were recruited through a convenient sampling technique.

Sampling

Both male and female patients with chronic hepatitis C infection aged 20 to 60 years were enrolled. Fixed-dose combination tablets consisting of 400 mg sofosbuvir and 100 mg velpatasvir were administered orally to all patients for 12 weeks. In addition, weight-based ribavirin was added to the combination of sofosbuvir and velpatasvir in case of liver cirrhosis. Hemoglobin in cirrhotic patients was optimized before commencing them on ribavirin and in cirrhotic patients with low hemoglobin despite optimizing with supplements, low dose ribavirin (600 mg daily) was used. Patients were stratified according to liver status (cirrhotic versus non-cirrhotic).

Diagnosis of chronic hepatitis C was based on the detection of HCV RNA fragments by reverse transcription-polymerase chain reaction (RT-PCR) in the hospital laboratory. Persistence of HCV RNA level more than 50 copies on RT-PCR for more than six months was labeled chronic HCV infection. Liver status was determined based on (1) stigmata of chronic liver disease on physical examination, including palmar erythema, jaundice, spider nevi, ascites, contractures, and loss of axillary and pubic hair; (2) laboratory tests like deranged liver function tests including serum albumin less than 3.5 gm/dl and international normalized ratio (INR) more than 1.2 or prothrombin time (PT) more than 15 seconds; and (3) imaging modalities, e.g., ultrasound showing shrunken liver, irregular margins, and coarse echotexture. The presence of all of the above was considered positive for liver cirrhosis. In the absence of the above findings, patients were labeled chronic HCV without cirrhosis. Patients with hepatocellular carcinoma, concurrent hepatitis B infection, history of the human immunodeficiency virus (HIV), history of a liver transplant, and evidence of resistance to sofosbuvir were excluded.

Data collection and analysis

Patients with chronic HCV infection, treatment naïve, were recruited from the hepatitis clinic and indoor medicine department, Khyber Teaching Hospital, Peshawar. Informed consent was taken from all study participants. Data regarding patients' parameters including age, gender, disease duration, and BMI were recorded before commencing treatment. All patients went through a detailed history and medical examination followed by necessary laboratory investigations including hemoglobin level, platelets count, serum bilirubin, serum albumin, PT, INR, and viral load. We also calculated the Child-Pugh score and Model for End-stage Liver Disease (MELD) score for all cirrhotic patients. Liver health was determined (cirrhotic versus non-cirrhotic) based on the criteria mentioned above.

The patients were monitored with weekly follow-up consisting of physical examination and routine labs till 12 weeks. Sustained virologic response at 12 weeks (SVR12) after treatment was used as an indicator of efficacy. RT-PCR was performed 12 weeks post-treatment, and patients were considered to have achieved SVR12 or responders if HCV viral load was undetectable or lower than 50 IU/ml. Failure to achieve SVR12 was labeled as treatment failure/non-responders. Adverse events were grouped into no complaints, mild, moderate, and severe. We labeled adverse events as mild if they were transient and not severe enough to require hospitalization or treatment interruption. For instance, nausea, vomiting, anorexia, headache, and epigastric pain were labeled as mild adverse effects. Moderate adverse events included worsening from the baseline condition, including worsening of Child-Pugh score, MELD score, liver function tests, and derangement in renal profile. Death as a result of treatment was labeled as a severe adverse reaction when other causes of death have been ruled out. Data regarding patients’ demographics, laboratory parameters, SVR12, and adverse events were collected and then analyzed using SPSS, version 22 (IBM SPSS Statistics, Armonk, NY).

Study endpoint

The study's primary endpoint was the determination of the rate of SVR12 after treatment and comparison among patients with and without cirrhosis. Another primary safety endpoint was the determination of adverse events experienced by the patients during the course and comparison among patients with and without cirrhosis.

## Results

A total of 65 patients with chronic HCV with cirrhosis and 58 patients without cirrhosis were enrolled. Among patients with cirrhosis, four were lost in the follow-up; hence, they were excluded. Three patients had experienced worsening of liver functions, renal functions, MELD score, and Child-Pugh score for two consecutive weeks after three weeks of initiation of treatment; hence, treatment was discontinued. Though they were followed, treatment was not reinitiated; hence, they could not be included in the study pool to determine SVR12. Therefore these three patients were also excluded. Eventually, a total of 58 patients with cirrhosis and 58 patients without cirrhosis were followed and studied.

The mean age and hemoglobin of patients with cirrhosis were greater than patients without cirrhosis. Tables 1, 2 depict the baseline characteristics of patients with cirrhosis and without cirrhosis, respectively.

**Table 1:** Baseline characteristics of patients with cirrhosis. BMI: body mass index; HCV: hepatitis C virus; HB: hemoglobin; ALT: alanine transaminase; INR: international normalized ratio.

	Minimum	Maximum	Mean	Std. deviation
Age (years)	31	57	44.84	8.108
BMI (kg/m^2^)	21.2	25.3	23.474	1.2338
Disease duration	10	36	21.64	7.417
HCV copies (IU/ml)	403,100	3,000,000	759,221.72	460,196.131
HB (gm/dl)	7.9	10.3	9.190	0.7813
Platelet count (per ml)	65,300	98,000	83,524.48	10,846.313
Serum albumin (gm/dl)	2.2	3.7	2.972	0.4595
Bilirubin (mg/dl)	1.6	2.9	2.219	0.4269
ALT (IU/L)	74	98	84.17	6.508
INR	1.7	2.4	1.912	0.1846

**Table 2:** Baseline characteristics of patients without cirrhosis. BMI: body mass index; HCV: hepatitis C virus; HB: hemoglobin; ALT: alanine transaminase; INR: international normalized ratio.

	Minimum	Maximum	Mean	Std. Deviation
Age (years)	22	57	37.38	9.860
BMI (kg/m^2^)	20.0	25.9	23.100	1.6910
Disease duration (months)	6	18	10.88	3.934
HCV Copies (IU/ml)	10,600	7,942,400	898,871.55	1,846,851.482
HB (gm/dl)	9.7	14.5	11.828	1.4224
Platelets count (per ml)	113,900	249,500	179,436.21	34,558.426
Albumin (gm/dl)	3.4	4.2	3.840	0.2463
Bilirubin (mg/dl)	0.6	1.2	0.938	0.1872
ALT (IU/L)	25	98	65.36	19.294
INR	0.7	1.3	1.014	0.1407

Efficacy

The efficacy of the sofosbuvir and velpatasvir combination was 98.3% in patients without cirrhosis as shown in Table 3 and 89.7% in patients with cirrhosis as shown in Table 4.

**Table 3:** SVR12 of patients without cirrhosis. SVR12: sustained virologic response at 12 weeks post-treatment.

SVR12	Frequency	Percent
Achieved	57	98.3
Not achieved	1	1.7
Total	58	100.0

**Table 4:** SVR12 of patients with cirrhosis. SVR12: sustained virologic response at 12 weeks post-treatment.

SVR12	Frequency	Percent
Achieved	52	89.7
Not achieved	6	10.3
Total	58	100.0

Among the non-cirrhotic cohort, only one patient (1.7%) could not achieve sustained virologic response (SVR) while in the cirrhotic cohort, six patients (10.3%) were non-responders. Subgroup analysis of patients who could not achieve SVR12 among the cirrhotic group showed that four out of six (66.66%) patients were in the age group of 41-60 years, and the majority of them were male participants (five patients, 83.3%). Five patients (83.3%) had a disease duration of more than 12 months, and five patients (83.3%) had hemoglobin less than 10 gm/dl. None of these subgroups' analyses showed statistical significance with respect to SVR12. Tables 5, 6 summarize the subgroup analysis of non-cirrhotic and cirrhotic patients, respectively.

**Table 5:** Subgroup analysis of patients without cirrhosis. SVR12: sustained virologic response at 12 weeks post-treatment; HB: hemoglobin.

		SVR12		Total	p-value
		Achieved	Not achieved		
Age	21-40 years	34 (97.1%)	1 (2.9%)	35 (100.0%)	0.414
	41-60 years	23 (100.0%)	0 (0.0%)	23 (100.0%)	
Disease duration	Less than 12 months	40 (97.6%)	1 (2.4%)	41 (100.0%)	0.516
	More than 12 months	17 (100.0%)	0 (0.0%)	17 (100.0%)	
HB	Less than 10 gm/dl	8 (88.9%)	1 (11.1%)	9 (100.0%)	0.019
	More than 10 gm/dl	49 (100.0%)	0 (0.0%)	49 (100.0%)	
Serum albumin	More than 3.5 gm/dl	13 (100.0%)	0 (0.0%)	13 (100.0%)	0.588
	Less than 3.5 gm/dl	44 (97.8%)	1 (2.2%)	45 (100.0%)	
Gender	Male	35 (97.2%)	1 (2.8%)	36 (100.0%)	0.430
	Female	22 (100.0%)	0 (0.0%)	22 (100.0%)	
Platelet count	Less than 150000	8 (88.9%)	1 (11.1%)	9 (100.0%)	0.019
	More than 150000	49 (100.0%)	0 (0.0%)	49 (100.0%)	

**Table 6:** Subgroup analysis of patients with cirrhosis. SVR12: sustained virologic response at 12 weeks post-treatment; HB: hemoglobin.

		SVR12		Total	p-value
		Achieved	Not achieved		
Age	21-40 years	19 (90.5%)	2 (9.5%)	21 (100.0%)	0.877
	41-60 years	33 (89.2%)	4 (10.8%)	37 (100.0%)	
Gender	Male	29 (85.3%)	5 (14.7%)	34 (100.0%)	0.194
	Female	23 (95.8%)	1 (4.2%)	24 (100.0%)	
Disease duration	Less than 12 months	7 (87.5%)	1 (12.5%)	8 (100.0%)	0.829
	More than 12 months	45 (90.0%)	5 (10.0%)	50 (100.0%)	
HB	<10 gm/dl	39 (88.6%)	5 (11.4%)	44 (100.0%)	0.652
	>10 gm/dl	13 (92.9%)	1 (7.1%)	14 (100.0%)	
Serum albumin	Less than 3.5 gm/dl	44 (89.8%)	5 (10.2%)	49 (100.0%)	0.935
	More than 3.5 gm/dl	8 (88.9%)	1 (11.1%)	9 (100.0%)	
Platelet count	Less than 75,000	12 (85.7%)	2 (14.3%)	14 (100.0%)	0.578
	More than 75,000	40 (90.9%)	4 (9.1%)	44 (100.0%)	

Safety

The incidence rate of adverse events among all the patients was 46.5% and most of the adverse events were mild. Overall, patients in the cirrhotic cohort experienced more adverse events (63.8%) than the non-cirrhotic cohort (29.3%). The most common mild complaints stated by study participants were gastrointestinal (GI) upsets, including pain epigastrium (46.2%) followed by fatigue (33.9%) and 19.9% developed miscellaneous adverse events such as headaches, rash, insomnia. Only 13 patients (22.4%) experienced moderate adverse events in the form of a slight elevation in Child-Pugh Score from the baseline, derangements in the liver and renal profile, or development of anemia. Still, that did not lead to the discontinuation of treatment. No death was observed as a result of treatment. No severe adverse events were recorded. Tables 7, 8 summarize the adverse events experienced by non-cirrhotic and cirrhotic patients.

**Table 7:** Adverse events in patients without cirrhosis.

Adverse events	Frequency	Percent
No complaints	41	70.68
Mild	15	25.86
Moderate	02	3.44
Severe	0	0

**Table 8:** Adverse events in patients with cirrhosis.

Adverse events	Frequency	Percent
No complaints	21	36.2
Mild	24	41.37
Moderate	13	22.4
Severe	0	0

## Discussion

For years, interferon-based therapy was administered for the treatment of chronic HCV infection. Besides poor efficacy, the regimen was complex and had several safety concerns. The introduction of DAAs was a landmark in the treatment of chronic HCV. The second-generation DAAs, including sofosbuvir (NS5B inhibitor) and velpatasvir (NS5A inhibitor), addressed all the limitations associated with the previous chronic HCV treatment modalities [9,10].

In our study, the sofosbuvir and velpatasvir combination was found highly effective against chronic HCV in patients with and without cirrhosis. SVR12 in patients with cirrhosis and without cirrhosis group was 89.7% and 98.3%, respectively. A study conducted by Wong et al. in Asia to determine the efficacy of sofosbuvir and velpatasvir combination with or without ribavirin in patients with chronic hepatitis C reported an overall SVR rate of 99.5%; however, the SVR rate in patients with decompensated cirrhosis was 88%. The study showed trends of SVR similar to our study [11]. Similarly, in a prospective observational study conducted in Pakistan in which 1,388 patients with chronic HCV were enrolled, and 30% of these patients received sofosbuvir and velpatasvir, the overall SVR rates for the cohort who received sofosbuvir and velpatasvir was 94.7, while in patients with cirrhosis, it was 88%. The findings of this study are in agreement with our study [12]. Our study showed relatively low SVR rates in patients with cirrhosis, 89.7% compared to 98.3% in non-cirrhotic chronic hepatitis C patients. Similar trends have been observed in other studies [11,12].

Results of our study with respect to the efficacy of the sofosbuvir and velpatasvir combination are similar to those reported by the ASTRAL trial, which reported 89% to 100% efficacy in patients with decompensated cirrhosis [13]. However, the results in our study in patients without cirrhosis are a little higher than those reported by Butt et al. in a study conducted at another center in Pakistan, which reported 92.5% efficacy in patients without cirrhosis [14]. A phase three trial by Esteban et al. on genotype 3 chronic HCV with cirrhosis reported a 95% SVR12 in patients without cirrhosis and 91% in cirrhotic patients, which is comparable to the results of our study [15]. Irrespective of the HCV genotype and cirrhosis status, Buggisch et al. reported a 99% rate of SVR12 in patients with chronic HCV infection, treated with sofosbuvir and velpatasvir combination regimen [16].

It is essential to provide appropriate attention to the safety profile of the study participants, particularly in patients with advanced liver fibrosis. In our study, the sofosbuvir and velpatasvir combination was considerably safe both in patients with and without cirrhosis. Only three patients with advanced-stage liver disease showed deterioration of liver functions and a rise in Child-Pugh score accompanied by progressive worsening of renal functions leading to discontinuation of study drugs. Such events were reported in a Japanese study as well. However, the adverse effects were not solely attributed to the study drugs considering the advanced stage of the liver disease also [17]. The majority of the adverse events recorded by the study participants in both groups were mild in nature, including gastrointestinal upsets followed by fatigue and miscellaneous complaints like headache and insomnia. Similar observations were reported by Charatcharoenwitthaya et al. in their study as well. However, no alarming adverse events were recorded [18]. In our study, 46.5% of patients experienced adverse events; however, most of the adverse events were mild and did not require any dose alteration. It is pertinent to mention that cirrhotic patients experienced adverse events more frequently (63.8%) than non-cirrhotic patients (29.3%). Similarly, the study conducted by Mei et al. to assess the safety and efficacy of sofosbuvir and velpatasvir reported a 44.4% incidence of adverse events. However, in this study, the most common adverse event was fatigue, while in our study, gastrointestinal upsets (46.2%) were more commonly reported, followed by fatigue (33.8%) [19].

Our study has limitations, including small sample size, the descriptive nature of the study, and lack of access to check resistance-associated substitution before treatment. Therefore, randomized trials with a large sample size are needed in our population to provide more accurate data about the safety and efficacy of sofosbuvir and velpatasvir in patients with chronic HCV.

## Conclusions

The combination of sofosbuvir and velpatasvir is highly effective against chronic HCV infection, both in patients with and without cirrhosis, irrespective of the genotype of HCV. However, the efficacy of this combination is slightly high in non-cirrhotic patients (98.3%) compared to cirrhotic patients (89.7%). Although the combination regimen of sofosbuvir and velpatasvir is extremely safe, mild adverse events such as gastrointestinal upsets, fatigue, and headaches are common, and the overall incidence of adverse events was 46.5% among all study participants. However, cirrhotic patients experienced adverse events more frequently than non-cirrhotic patients. Most of the adverse events were mild and in all cases, no alterations in dose or drugs were required. Based on this study, the authors recommend that the combination of sofosbuvir and velpatasvir is safe and efficacious and should be offered to all patients with chronic hepatitis C irrespective of their liver status and genotype.
